# Ensuring access to high-quality resources reduces the impacts of heat stress on bees

**DOI:** 10.1038/s41598-019-49025-z

**Published:** 2019-08-29

**Authors:** Maryse Vanderplanck, Baptiste Martinet, Luísa Gigante Carvalheiro, Pierre Rasmont, Alexandre Barraud, Coraline Renaudeau, Denis Michez

**Affiliations:** 1 Laboratory of Zoology, Research Institute for Biosciences, University of Mons, Place du Parc 23, 7000 Mons, Belgium; 2 Evo-Eco-Paleo - UMR 8198, CNRS, Université de Lille, F-59000 Lille, France; 3 Departamento de Ecologia, Universidade Federal de Goiás, Campus Samambaia, Goiânia, GO Brazil; 4 Center for Ecology, Evolution and Environmental Changes (cE3c), University of Lisboa, Lisbon, Portugal; 5 Pierre and Marie Curie University, Paris-Sorbonne 4, Place Jussieu, 75005 Paris, France

**Keywords:** Climate-change ecology, Conservation biology

## Abstract

Pollinators are experiencing declines globally, negatively affecting the reproduction of wild plants and crop production. Well-known drivers of these declines include climatic and nutritional stresses, such as a change of dietary resources due to the degradation of habitat quality. Understanding potential synergies between these two important drivers is needed to improve predictive models of the future effects of climate change on pollinator declines. Here, bumblebee colony bioassays were used to evaluate the interactive effects of heat stress, a reduction of dietary resource quality, and colony size. Using a total of 117 colonies, we applied a fully crossed experiment to test the effect of three dietary quality levels under three levels of heat stress with two colony sizes. Both nutritional and heat stress reduced colony development resulting in a lower investment in offspring production. Small colonies were much more sensitive to heat and nutritional stresses than large ones, possibly because a higher percentage of workers helps maintain social homeostasis. Strikingly, the effects of heat stress were far less pronounced for small colonies fed with suitable diets. Overall, our study suggests that landscape management actions that ensure access to high-quality resources could reduce the impacts of heat stress on bee decline.

## Introduction

Biotic pollination is essential for sustaining plant communities^1^ and is also an important ecosystem service^2^, which is threatened by the ongoing global decline of pollinators^3^. Bumblebees, a group of pollinators particularly important in temperate and arctic climatic regions^4^, are highly vulnerable to climatic^5,6^ and nutritional stresses^7^ driven by the transformation of diverse landscapes into large agricultural monocultures^8^, among other threats such as pesticide exposure and habitat fragmentation^9^. As for most bees, bumblebees rely exclusively on floral pollen and nectar for their nutrition, and diet suitability (i.e. amino acid content, sterols and protein:lipid ratio) can impact bumblebee offspring number, colony size, mortality and immunity^7,10–12^. Moreover, the loss of a preferred host-plant can induce starvation and developmental delay in bumblebee colonies^13,14^. Climate change might increase the probability of losing preferred floral resources by changing phenologies and distributions and creating mismatches between bees and their resources^15,16^, or by changing the quality and quantity of their floral resources^17^. In addition, the expected increase in the intensity and the frequency of extreme events such as heat waves^18^ can affect physiology and increase insect mortality (e.g. due to ontogenic development, changes water balance, fertility and immunity)^19,20^, potentially affecting the ability to detect suitable resources. Moreover, the lack of a suitable diet might decrease the resilience of organism facing heat wave in a similar manner to the stress of pesticide exposure^21^. Therefore, it is expected that any negative impact caused by heat stress will be more accentuated when bees are also subjected to nutritional stress. However, it is still unclear if heat and nutritional stresses influence the effects of each other^22^. A better understanding on how these main drivers of change affect bees is essential for the development of appropriate public policies and conservation plans.

Some ecological traits like sociality can mitigate environmental stresses. For bumblebees, the number of workers in a colony shapes its development and depends on both phenology and species. While arctic bumblebees (e.g. *B. alpinus*, *B. polaris*) are known to make colonies with a very small number of offspring (40–50)^23^, tropical species build huge nests with numerous workers, the largest recorded colony belonging to *B. transversalis* with more than 3,000 individuals^24^. The number and the size of workers not only influence brood nest development, nest maintenance and feeding of larvae^25^, but also food collection and thermal sensitivity, with small colonies likely to be more sensitive to extreme temperature variations^25–29^. Considering the size of colonies of social insects is hence essential to evaluate how sociality can buffer environmental stresses.

**Figure 1:**
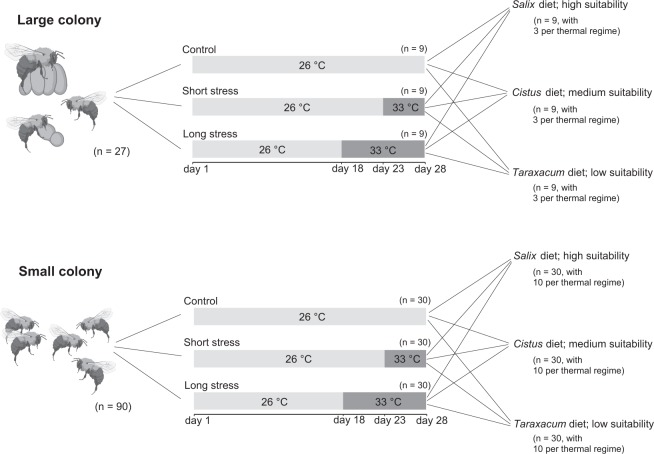
Experimental design. From a total of 117 colonies, one third were reared at a normal temperature (26 °C, control). The remaining colonies were divided in two groups, and exposed to climatic stress (33 °C) during five (short stress) or ten (long stress) days. Colonies were fed for 28 days on diets with a dominance of *Salix* pollen (high suitability), *Cistus* pollen (medium suitability) or *Taraxacum* pollen (low suitability). Mortality, offspring production and resource collection (i.e. pollen and syrup) were monitored during or at the end of the bioassays.

Interactive effects between climate and floral resources have rarely been addressed^30^ but are important to consider given their implications and relevance to global change, especially under future climatic scenarios^18,31^. To address these knowledge gaps, we used the buff-tailed bumblebee (*Bombus terrestris*) as a model organism and designed a fully crossed experiment (Fig. 1) to test the effect of (i) three distinct pollen diets displaying different amino acid concentrations and sterolic composition (i.e. low, medium and high suitability); (ii) three thermal regimes (i.e. control, short and long climatic stress); and (iii) two colony sizes (i.e. small and large colonies). This experiment is only possible with species that are manageable and we consider *B. terrestris* to be a better choice in comparison to *Apis mellifera* since this species is still present into the wild and its management started only recently (so less impact on genetics). *Bombus terrestris* is a robust and widespread heterothermic bee native to Europe (Euro-Mediterranean distribution) with the ability of endothermy, and is probably among the most well adapted bumblebee species to warm and dry conditions with a high resilience to extreme events^32,33^. However its ability to regulate its internal body temperature is limited, which makes individuals sensitive to climate change including heat waves that have become more frequent across its native range in recent decades^34^ and are likely to intensify in frequency and amplitude^18^. *Bombus terrestris* is a primitively eusocial bee and while it can produce large colonies with more than 100 workers, its colonies contain only some individuals at the beginning of their development^35^. This species displays a considerable flexibility in the seasonal timing of colony development (e.g. summer aestivation or multi-voltinism) and in their floral choices^36,37^. However, colonies do not show equal development on all pollen diets, with for instance diets with a dominance of Asteraceae pollen increasing larval mortality and decreasing individual offspring mass^38^. All these features make *B. terrestris* an appropriate pollinator model to assess the individual and combined effects of nutritional and heat stresses considering sociality. We expect that low suitability diets and long periods of heat stress will negatively affect colony performance, and that these two effects will act interactively, with large colonies being less affected than small ones.

## Results

Performance of bumblebee colonies as well as their feeding responses have been evaluated based on colony growth (i.e. total mass gain of the nest), composition of brood (i.e. eggs, non-isolated larvae, isolated larvae and pupae), mortality, total pollen and syrup collection (i.e. mass of pollen and syrup consumed and stored) (see Methods section for details). Overall our results show that while both nutritional and heat stress reduced colony development, there are important interactive effects between these two drivers of bee decline (Tables S1 and S2).

**Figure 2:**
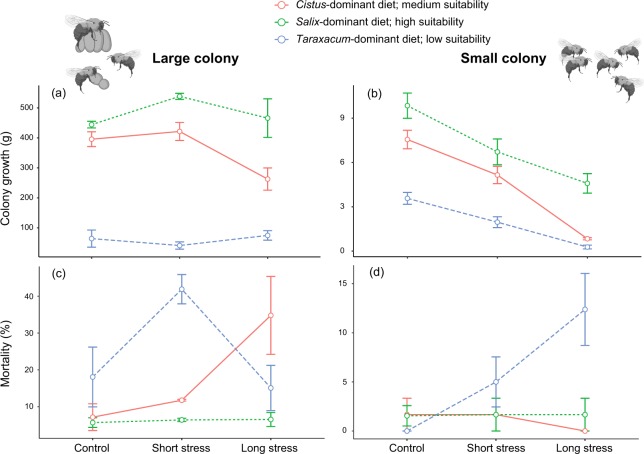
Colony development. Colony growth (**a**,**b**) and mortality (**c**,**d**) for large (left) and small (right) colonies exposed to different levels of environmental stresses (mean ± SE). Diet with a dominance of *Salix* sp. is highly suitable, diet with a dominance of *Cistus* sp. has medium suitability, and diet with a dominance of *Taraxacum* sp. has low suitability. Statistics are reported in Table S1.

**Figure 3:**
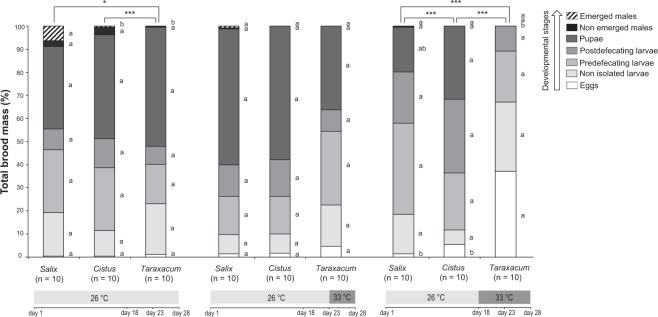
Small colony dynamics. Brood composition at different developmental stages expressed as percentage of total brood mass (i.e. dynamics of micro-colony development) for small colonies exposed to different levels of environmental stresses. Diet with a dominance of *Salix* sp. is highly suitable, diet with a dominance of *Cistus* sp. has medium suitability, and diet with a dominance of *Taraxacum* sp. has low suitability. Asterisks indicate significant differences in brood composition between micro-colonies fed different pollen diets (pairwise perMANOVAs; *p < 0.05; **p < 0.01; ***p < 0.001). Different letters indicate significant differences in the proportion of brood stages among bioassays (post-hoc tests, p < 0.05).

**Figure 4:**
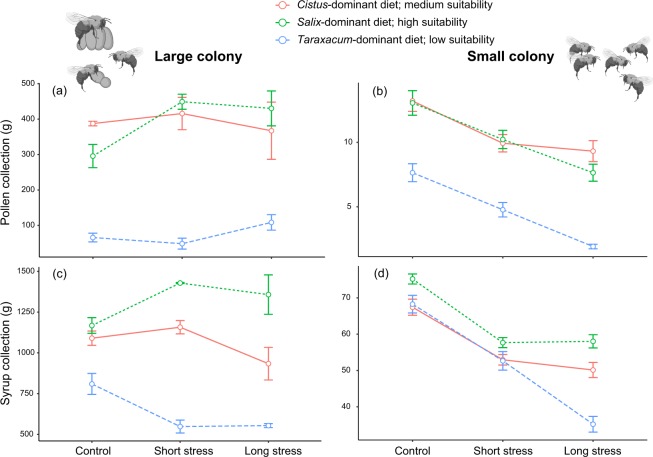
Resource collection. Pollen collection (**a**,**b**) and syrup collection (**c**,**d**) for large (left) and small (right) colonies exposed to different levels of environmental stresses (mean ± SE). Diet with a dominance of *Salix* sp. is highly suitable, diet with a dominance of *Cistus* sp. has medium suitability, and diet with a dominance of *Taraxacum* sp. has low suitability. Statistics are reported in Table S2.

For both colony sizes (i.e. large and small), colony growth (Fig. 2a,b) and mortality (Fig. 2c,d) were significantly affected by nutritional stress (see Table S1 for statistical details). Moreover, the dissections of small colonies highlighted a reduction of male production in colonies that fed on low suitability diets (the *Taraxacum*-dominant diet), indicating a slowing down in brood development (Fig. 3). When colonies had only access to low suitablity diets (i.e. the *Taraxacum*-dominant diet compared to the *Salix* and *Cistus*-dominant diets), the collection of both pollen (Fig. 4a,b) and syrup (Fig. 4c,d) significantly decreased. However, for small colonies the negative effects of nutritional stress became more accentuated when colonies were subjected to longer heat stress, especially for syrup collection (Fig. 4d) (see Table S2 for statistical details).

Heat stress significantly reduced colony growth, but this effect was much more accentuated in small colonies, where negative effects were seen at low to medium stress (Fig. 2b). When fed a high quality diet (i.e. *Salix*-dominant diet) the impacts of increasing heat stress from medium to high were less pronounced for both small and large colonies (Fig. 2a,b) (see Table S1 for statistical details). Effects on mortality were more variable, but for both small and large colonies, when fed a high quality diet the effects of heat stress were stable, while under a low quality diet mortality varied greatly in large colony (Fig. 2c) and tended towards increasing in small ones (Fig. 2d) despite non-significant pairwise comparisons (see Table S1 for statistical details). As for nutritional stress, heat stress also affected colony dynamics, slowing down brood development of small colonies (especially if fed on lower quality resources) by reducing male production and increasing the relative importance of eggs mass, regardless of the stress duration (Fig. 3). Regarding feeding behavior, large colonies were substantially more resilient than small colonies. Small colonies showed pronounced declines in both pollen and syrup collection when stress changed from low to medium intensity (Fig. 4b,d), while large colonies (under no or little nutritional stress) increased pollen and syrup collection when heat stress changed from low to medium, and only reduced resource collection after heat stress was raised from medium to high (Fig. 4c). Strikingly, when bees were subjected to a low quality diet, the impacts of heat stress on syrup collection became more pronounced for both small (Fig. 4d) and large colonies (Fig. 4c), with declines already detected for this last group when stress was raised from low to medium (see Table S2 for statistical details).

## Discussion

Although many studies have evaluated the effects of climate, land-use and other environmental changes on bumblebees^6,39^, combined effects among them have rarely been addressed^30^. Moreover, despite the recognized importance of these factors for bee population viability, the lack of controlled experiments limits our knowledge to understand how each factor contributes to their ongoing decline. Our study reveals important effects of heat stress and changes in bee diet under controlled and reproducible laboratory conditions, and also highlights striking combined negative effects of environmental stresses on colony development. However more studies are needed to better understand the mechanistic causes of *B. terrestris* response to heat stress. Below we discuss the implications of our findings related to the isolated and combined effect of each type of stress.

### Nutritional stress

Here we show that changes in diet quality can have important impacts on colony development and impact both pollen and syrup collection by bumblebee workers. These findings strengthen the idea that nutritional stresses caused by unfavourable properties of pollen sources (e.g. secondary metabolites of Asteraceae pollen) affect bumblebee colonies performance^40^ as already shown for a wide variety of bee species (e.g. the Western honeybee *Apis mellifera*^41^, some solitary bee species of the Megachilidae family^42,43^).

Such behavioral changes in resources collection (reduction in bee visits) could lead to a reduction in pollinator population size, as well as impact the pollination of wild plants and crops by reducing the frequency of bee visits, one of the most important variables for determining plant reproductive success^44–47^.

In current conservation strategies, one main approach to mitigate natural habitat fragmentation is the creation of wildflower strips to maintain pollinator networks^48^. Plant mixes are selected to maximize the number of bee species for a fixed cost^49^. Our results suggest that these commercial seed mixtures should be optimized to provide a suitable diet to pollinators (i.e. generalist and specialist bees) for preserving their health and development, based on ecological constraints such as the nutritive quality of floral resources and not only on human and financial considerations^49^.

### Heat stress

Predictive models show that the frequency of extreme climatic events (e.g. heat waves) will increase by the 2040 s in North America and Europe^18,50^, including in relatively hot areas such as Mediterranean climate regions (part of the native distribution of *B. terrestris*)^51,52^. The intensity and duration of these events will also become higher with a more widespread effect^18,53,54^, jeopardising ecological systems^55,56^. Our results clearly show that such future climatic changes are likely to cause significant negative impacts on bumblebee colonies. The fact that we found a delay in the development of small colonies under long heat stress as well as a low colony growth (Figs 3b, 4) could be due to physiological disruption to bees at several development stages^57^. While adult bees can tolerate a large range of temperatures, their ability to regulate brood nest temperature (which is crucial for the larval development^32^) can be reduced when exposed to high temperatures^58^. As the percentage of workers required for this task (e.g. via wing fanning) increases, fewer bees are available to ensure maintain the nest, feed the larvae and collect pollen and nectar^59^. High temperatures, even for a short time, could disrupt thermoregulation and could have a negative effect on colony development^58^, which could explain the results found for small colonies (Figs 3b, 4). Bees can compensate by endothermic heat production, evaporation of water or wing fanning^32,58^, but such behavior incurs a substantial ergonomic cost. Such recruitment of workers for buffering high temperature could partly explain the observed decrease in resources collection in the small and large colonies bioassays (see Fig. 2a–d). In addition, despite such efforts, workers were still not able to maintain the brood temperature within the optimal range (28–32 °C). Such effect could be related to environmental stress and/or bacterial development. Overall, our data show that heat exposure represents a non-negligible risk for the survival of colonies and maintenance of pollinator populations.

In Europe, ALARM climatic scenarios describe a rise of the mean annual temperature from 3.0 °C to 6.1 °C by the end of the 21^st^ century^31^. Such future climatic scenarios generally do not consider heat waves because of the challenge of modelling these accurately^60^. Our findings highlight the importance of improving prediction of heat waves to better understanding the impacts of climate change on bees, pollination and productivity of pollinator dependent crops.

Not all bee species are equally vulnerable to climatic changes^5^. Our focal species, *B. terrestris*, is thought to have a particularly good tolerance to environmental stresses^33^, which is usually associated with invasive potential to the detriment of native species^61^. It is therefore possible that other bee species are more susceptible to the effects of environmental stress addressed here. Species with naturally smaller colonies and species that evolved in thermally stable environments (i.e. that do not evolve mechanisms of thermoregulation) may be particularly susceptible. Future experimental studies involving a larger set of species would improve our ability to predict impacts of environmental changes at the community level.

### Colony size

The difference between the observed resilience of large and small colonies is likely related to *B. terrestris* social buffering abilities. The mechanism highlighting the regulation of thermic homeostasis of colonies has been well studied within honeybees^62^, but the physiological effect of sociality on thermoregulation and its costs still remain poorly investigated^63^. We may assume that the division of labor among foraging, brood maintenance and fanning tasks is more problematic in a small colony than in a large one with a greater number of available workers. Moreover, colonies with only some workers may not be able to maintain brood temperature when the air temperature is higher than 32 °C^58,64^, compromising optimal brood development. This suggests that nutritional and/or heat stresses in early spring (i.e. when young colonies are growing and have few workers) might cause higher negative impacts than at the end of the summer (i.e. when colonies are larger with more numerous workers). In the same way, such impacts might be more severe for bumblebee species that build smaller colonies, such as boreal-alpine species like *B. monticola* or *B. alpinus*^5^ which are likely to experience severe climate changing in their native ranges^65^.

### Combined effects

One of the most striking results of this study is that the effects of exposure to heat waves were less intense when bees had access to a high quality diet (i.e. *Salix* diet) with colony growth and feeding behavior being less impacted for small colonies fed on this high quality diet. Although our experiment did not provide clear evidence for bumblebee colony death under heat stress, under the best scenario (i.e. a recovery after heat wave) the developmental delay of the colony could increase the phenological mismatch between plants and pollinators^66^ affecting both partners^67,68^, as well as decreasing the colony size, thereby affecting the number of workers and therefore susceptibility to further environmental stress^26,69,70^. Such consequences may be worsened depending on the surrounding plant species available for bumblebees forage (i.e. combined effects). In field conditions, the synergistic depression resulting from heat stress and diet suitability might occur during a drought-related heat wave episode with a water deficit^19,71^. These events are expected to become more frequent^18^ and can decrease floral resources and/or cause a phenological drift. Consequently, the performance of bumblebee colonies (especially in arcto-alpine regions) and, bee-flower interactions could be dramatically impacted^65,72,73^.

While the ideal scenario is that humanity as whole substantially reduces carbon emissions (green-house gas emissions decline after 2020)^74^, the most optimistic green-house gas concentration trajectory (Representative Concentration Pathway RCP 2.6) still leads to a slight decay of heat waves after a half-century of increase^75^. Therefore, it is essential to improve land use management to minimize the impacts on bees and associated ecosystem services. As previously discussed, flower strips are one of the common practices aiming to minimize pollinator loss^48^, but caution has to be paid to species selection in the plant mixes. Although the quality of resources is essential^76^, it is also important to promote a sufficient diversity to cope with physiological requirements of a wide range of bees^77^, and to allow pollen mixing behavior^78^. Indeed mutliforal diets are known to ensure optimal nutritional requirements for generalist bees (i.e. mitigation of unfavourable pollen properties)^78,79^ and may also improve immune system of bees (e.g. *Apis* *mellifera*)^80,81^. Both criteria should then be considered for bee conservation management.

## Concluding remarks

Based on a fully crossed experiment in controlled conditions, our findings highlight the importance of having suitable host plants for social generalist bees during extreme climatic events, instead of simply increasing floral resources (as suggested at a landscape level by previous studies^82^). The next step would be to evaluate bee health in landscapes with low and high quality of resources in different climatic regions, which would require consequent investment but allow for overcoming the lab-based approach.

It is important to highlight that climate change encompasses not only temperature changes but changes in precipitations and humidity levels. Studies that address the combined effects of changes in multiple climatic variables would be important. Moreover other environmental threats may have further interactive effects. For example, pesticides are known to depress thermoregulation in honeybees^83^ and synergistic effects between pesticide exposure and nutritional stress have recently been highlighted^84^. Human driven changes in biogeochemical flows have also been substantial^85^, and those may change the chemical content of flower resources^40^. Such changes in nectar and pollen amino acid and sugar compositions may lead to a higher mortality rate in bumblebee colonies^40^.

Overall, our findings highlight the importance of considering a large range of threats, to cope with the reality of the ongoing worldwide bee decline. Future studies investigating single and combined effects of climate, land-use changes and other environmental drivers on bee populations are essential. In addition, our focal species, *Bombus terrestris* is a ubiquitous, generalist and resilient species^33^, and it is likely that these effects could be more severe for rarer and more sensitive bumblebee species. Therefore, species traits may play an important role, and considering how different bee species (e.g. sensitivity, resilience and adaptive capacity)^86^ react to such changes can help predict impacts of ongoing environmental changes.

## Methods

### Experimental design

The fully crossed experiment was performed under carefully controlled and reproducible laboratory conditions. Although bumblebee workers did not forage freely, in comparison to a field experiment, this laboratory experiment allowed for a greater control of extern and explicative variables (i.e. diet quality and temperature), and permits to draw reliable conclusions on causal relationships.

#### Colony size

We considered two sizes of colonies: large colonies (queen-right colonies) and small colonies (queen-less micro-colonies), which could be considered as a proxy for young wild colonies (i.e. early colony development starting just after solitary queen over-wintering). For large colonies, we used a total of 27 queen-right colonies of *Bombus terrestris* reared in plastic boxes (14 * 29 * 23 cm), which were initiated and standardized with 60 color-marked workers and one queen. For small colonies, we used 90 queen-less micro-colonies with five workers reared in plastic boxes (8 * 16 * 16 cm) (Fig. 1). Queen-less micro-colonies were generated by randomly selecting five workers of each of six queen-right colonies. No brood was provided. A hierarchical system occurred quickly in micro-colonies with a worker exerting its dominance on the others and laying male eggs so that they were used as a proxy of early stages of development of queen-right colonies^87^. All workers within a micro-colony originated from the same colony to avoid aggressive behavior. All micro-colonies started to produce their own brood after a few days. Colonies were provided by Biobest NV (Westerlo, Belgium). They were maintained in constant darkness, in a relative humidity of 60–65% and manipulated under red light to minimize disturbance^88^. Prior to the experimentation, colonies and sugar boxes have been weighed.

#### Nutritional stress

To assess the importance of nutritional stress, large and small colonies were fed on three pollen diets previously used in similar experiments^12,38^ and displaying different dominant plant species, and then different levels of suitability for generalist bumblebees: diet with a dominance of *Salix* sp. (known as a highly suitable diet^10,38,76,89^), diet with a dominance of *Cistus* sp. (known as a suitable diet^10,38,76^) and diet with a dominance of *Taraxacum* sp. (known as a poorly suitable diet^41,90,91^). Pollen of *Sali*x has been previously described as an excellent resource for *B. terrestris* colony development (18.6% of total amino acid content) while *Cistus* pollen had a rather negative impact on colony development (13.5% of total AA content). *Taraxacum* pollen is both chemically (e.g. lack in essential amino acids tryptophan, phenylalanine and arginine) and structurally unsuitable (i.e. thick multilayer pollen grains), which leads to constraints for bee development related to pollen nutritional content, toxicity and digestibility^38,42,43,79,90–92^. Pollen loads were purchased from the companies “Ruchers de Lorraine” for *Salix*-dominant diet and *Taraxacum*-dominant diet, and “Pollenergie France” for *Cistus*-dominant diet. They are sold as organic nutrition complement (i.e. free of pesticides). Prior to the experiment, blends of pollen loads were mixed with inverted sugar syrup (BIOGLUC, Biobest, also used for sugar resources) to obtain consistent candies stored at −20 °C. New pollen candy was provided every two days, while the previous one was removed at the same time before decaying and weighed to assess the pollen collection. The bumblebee workers were not allowed to forage outside the nest.

#### Heat stress

Considering a heat wave as a punctual and intense climatic extreme event, we have chosen a static thermo-tolerance method for this bioassay^5,93–95^ with a constant temperature mimicking hyperthermic stress. A heat wave can be defined as a period of five days or more, during which daily thermal maxima exceed the average local maximum by 5 °C^53^. To select the stress temperature in our experiment, soil temperature at 15 cm depth (i.e. usual depth of bumblebee nest^32^) was recorded in Belgium (Kalmthout, 51°24’N 04°24’E) every four hours from 23 July 2018 to 28 July 2018 (i.e. heat wave; KMI, 2018) using a data logger (Voltcraft DL-181 THP USB Ambient Monitoring Data Logger). Based on the results (Fig. S1), we used 33 °C as the stress temperature, which is the upper limit where bumblebees can thermo-regulate their colonies by ventilation^32,58^, and 26 °C as the control one^35^. We used three temperature treatments: (i) without stress (control group) at 26 °C during 28 days; (ii) short stress at 26 °C during 23 days and 33 °C during 5 days; (iii) long stress at 26 °C during 18 days and 33 °C during 10 days. A single room was used to avoid manipulation bias by moving some colonies during the experiment. The room was set at 26 °C for 28 days (control temperature) and then set at 33 °C for 10 days (stress temperature) (i.e. the total duration of the experiment was 38 days). The control group was introduced in the room at day 1 and removed at day 28; the short stress group was introduced in the room at day 5 and removed at day 33; and the long stress group was introduced in the room at day 10 and removed at day 38. Each temperature treatment was tested with the three diets and two colony sizes for assessing the effects of these environmental stresses on colony performance, considering the effect of colony size (Fig. 1).

### Assessed parameters

Based on the methodology from Taseï & Aupinel^10^, several parameters were used to estimate fitness and development of bumblebee colonies: (i) total pollen and syrup collection (i.e. mass of pollen and syrup consumed and stored), which influences brood production and larval development time^96^; (ii) colony growth (i.e. total mass gain of the nest), which can influence food provisioning, brood care, defense and the production of sexual^69,70,97,98^; and (iii) mortality (i.e. number of dead individuals divided by total individuals including initial ones and new-emerged ones), which affects workforce and hence foraging activities and brood care, as well as the ability to respond to environmental stresses such as temperature fluctuations^26^.

To assess the effect of environmental stresses on the colony development (i.e. occurrence of the different larval stages), all small colonies have been dissected to determine both mass and number of each individuals (i.e. brood composition) considering separately the different brood stages, namely eggs, non-isolated larvae, isolated and pre-defecating larvae, isolated and post-defecating larvae, pupae, non-emerged and emerged males, which was not possible for the large ones.

### Statistical analyses

We performed statistical comparative analyses of the colony performances using R environment^99^. Statistical analyses using generalized linear models (‘glm’ command in R-package stats) were conducted for large and small colonies to evaluate the effect of nutritional and heat stresses as well as their interaction. Post-hoc multiple comparisons were run using Tukey contrasts (‘glht’ function from R-package multcomp). Data on colony growth were not normally distributed, and were analysed assuming a gamma error distribution. Data on mortality were normally distributed for large colonies, but for small colonies mortality was a rare event, and was hence computed as binary variable (binomial distribution). Whereas the test compares the mortality rates among conditions for large colonies, it rather compares the probability of dying for small ones.

For the analysis of brood composition (relative mass of the different larval stages), we performed a permutational multivariate analysis of variance (perMANOVA) on arcsine-transformed data using the Bray-Curtis dissimilarity matrix and 1000 permutations (“adonis” command, R-package vegan^100^): it was led on the combined effect of both factors (3 * 3 levels). Prior to these tests, the multivariate homogeneity of within-group covariance matrices was verified using the “betadisper” function. When a significant difference was detected, we performed multiple pairwise comparisons with an adjustment of p-values (Bonferroni correction). In addition, separate two-way ANOVA and Tukey post-hoc tests were conducted to assess the effect of the environmental stresses on each developmental stage.

## Supplementary information

– Supplementary Information – Ensuring access to high-quality resources reduces the impacts of heat stress on bees
