# Supplementary material for: Ensuring access to high-quality resources reduces the impacts of heat stress on bees
Source: Sci Rep. 2019 Aug 29;9:12596. doi: 10.1038/s41598-019-49025-z (PMC6715733; doi:10.1038/s41598-019-49025-z)
Supplement: Supplementary file 1 — – Supplementary Information – Ensuring access to high-quality resources reduces the impacts of heat stress on bees [file 41598_2019_49025_MOESM1_ESM.pdf]

# – Supplementary Information –

Ensuring access to high-quality resources reduces the impacts of heat stress on bees

Maryse Vanderplanck[1,2,†,*] , Baptiste Martinet[1,†,*], Luísa Gigante Carvalheiro[3,4], Pierre Rasmont[1], Alexandre Barraud[1,5], Coraline Renaudeau[5], Denis Michez[1]

[1]Laboratory of Zoology, Research Institute for Biosciences, University of Mons, Place du Parc 23, 7000 Mons, Belgium

[2] Evo-Eco-Paleo - UMR 8198, CNRS, Université de Lille, F-59000 Lille, France

[3]Departamento de Ecologia, Universidade Federal de Goiás, Campus Samambaia, Goiânia - GO, Brazil

[4]Center for Ecology, Evolution and Environmental Changes (cE3c), University of Lisboa, Lisbon, Portugal

[5]Pierre and Marie Curie University, Paris-Sorbonne 4, Place Jussieu, 75005 Paris, France

[†] Maryse Vanderplanck and Baptiste Martinet have contributed equally to this work (co-first authors)

[*]*Corresponding authors:*

Maryse Vanderplanck, maryse.vanderplanck@umons.ac.be, +32 65 373436, postal address – Laboratory of Zoology, Research Institute for Biosciences, University of Mons, Place du Parc 23, 7000 Mons, Belgium

Baptiste Martinet, baptiste.martinet@umons.ac.be, +32 65 373466, postal address – Laboratory of Zoology, Research Institute for Biosciences, University of Mons, Place du Parc 23, 7000 Mons, Belgium

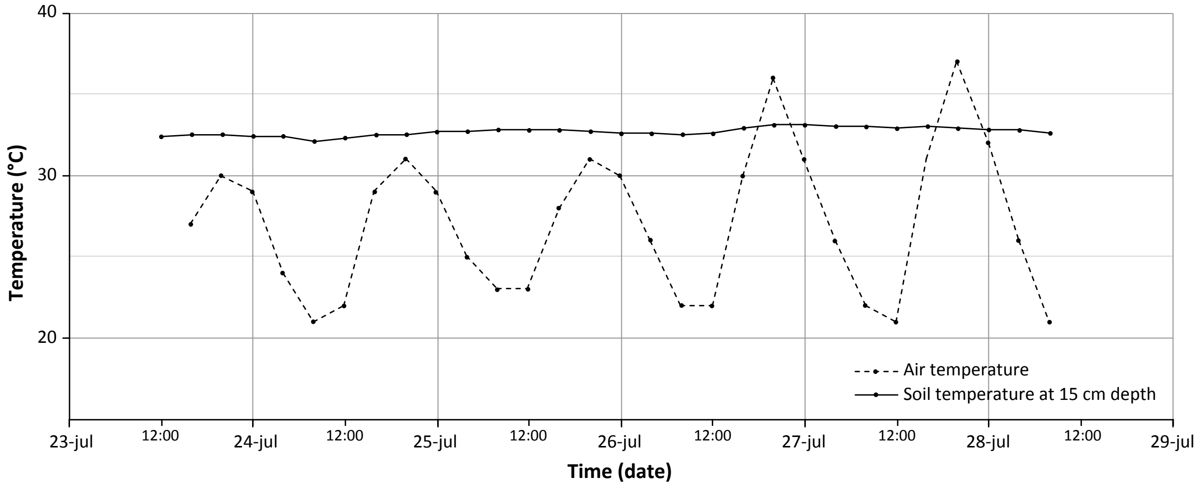

Figure S1. Outdoor air temperature and soil temperature at 15 cm depth in Belgium (Kalmthout) during a heat wave (July 2018). Soil temperature was measured using a data logger, air temperature was retrieved on www.timeanddate.com on January 2018.

Table S1. Performance of bumblebee colonies. Global effects of nutritional stress and heat stress as well as two-way interactions. Results of one-way analysis of variance (ANOVA) models and post-hoc tests are presented.

		**Colony growth (g)**<br>Model: Gamma<br>Link: inverse	**Mortality (%)**<br>Model: gaussian (large); binomial (small)<br>Link: identity (large); logit (small)
**Large colony**	Nutritional stress	$F_{2,18}$= 68.21<br>p< 0.001	$F_{2,18}$= 9.75<br>p = 0.0014
	Heat stress	$F_{2,18}$= 0.64<br>p= 0.5408	$F_{2,18}$= 3.03<br>p= 0.0734
	Nutritional stress : Heat stress	$F_{4,18}$= 1.63<br>p = 0.2105	$F_{4,18}$= 6.33<br>p= 0.0023
		Post-hoc tests	
	Control group – *Salix*-dominant diet	b	a
	Control group – *Cistus*-dominant diet	b	a
	Control group – *Taraxacum*-dominant diet	a	ab
	Short stress group – *Salix*-dominant diet	b	a
	Short stress group – *Cistus*-dominant diet	b	ab
	Short stress group – *Taraxacum*-dominant diet	a	c
	Long stress group – *Salix*-dominant diet	b	a
	Long stress group – *Cistus*-dominant diet	b	bc
	Long stress group – *Taraxacum*-dominant diet	a	ab
**Small colony**	Nutritional stress	$F_{2,81}$= 31.54<br>p< 0.001	χ^2= 6.19, df = 2<br>p= 0.0452
	Heat stress	$F_{2,81}$= 27.62<br>p< 0.001	χ^2 = 2.11, df = 2<br>p= 0.3478
	Nutritional stress : Heat stress	$F_{4,81}$= 19.89<br>p< 0.001	χ^2 = 11.10, df = 4<br>p= 0.0254
		Post-hoc tests	
	Control group – *Salix*-dominant diet	e	a
	Control group – *Cistus*-dominant diet	de	a
	Control group – *Taraxacum*-dominant diet	cd	a
	Short stress group – *Salix*-dominant diet	de	a
	Short stress group – *Cistus*-dominant diet	de	a
	Short stress group – *Taraxacum*-dominant diet	bc	a
	Long stress group – *Salix*-dominant diet	ce	a
	Long stress group – *Cistus*-dominant diet	b	a
	Long stress group – *Taraxacum*-dominant diet	a	a

Table S2. Resource collection of bumblebee colonies. Global effects of nutritional stress and heat stress as well as two-way interactions. Results of one-way analysis of variance (ANOVA) models and post-hoc tests are presented.

		Pollen collection (g)<br>Model: gaussian<br>Link: identity	Syrup collection (g)<br>Model: gaussian<br>Link: identity
**Large colony**	Nutritional stress	$F_{2,18}$= 67.21<br>p< 0.001	$F_{2,18}$= 87.67<br>p< 0.001
	Heat stress	$F_{2,18}$= 1.93<br>p= 0.1746	$F_{2,18}$= 1.88<br>p= 0.1808
	Nutritional stress : Heat stress	$F_{4,18}$= 1.90<br>p = 0.1540	$F_{4,18}$= 5.69<br>p= 0.0039
		Post-hoc tests	
	Control group – *Salix*-dominant diet	b	ce
	Control group – *Cistus*-dominant diet	b	cd
	Control group – *Taraxacum*-dominant diet	b	ab
	Short stress group – *Salix*-dominant diet	b	e
	Short stress group – *Cistus*-dominant diet	b	ce
	Short stress group – *Taraxacum*-dominant diet	a	a
	Long stress group – *Salix*-dominant diet	b	de
	Long stress group – *Cistus*-dominant diet	b	bc
	Long stress group – *Taraxacum*-dominant diet	a	a
**Small colony**	Nutritional stress	$F_{2,81}$= 68.26<br>p< 0.001	$F_{2,81}$= 25.75<br>p< 0.001
	Heat stress	$F_{2,81}$= 38.67<br>p< 0.001	$F_{2,81}$= 101.5307<br>p< 0.001
	Nutritional stress : Heat stress	$F_{4,81}$= 0.85<br>p = 0.4968	$F_{4,81}$= 7.44<br>p< 0.001
		Post-hoc tests	
	Control group – *Salix*-dominant diet	d	c
	Control group – *Cistus*-dominant diet	d	c
	Control group – *Taraxacum*-dominant diet	bc	c
	Short stress group – *Salix*-dominant diet	cd	b
	Short stress group – *Cistus*-dominant diet	c	b
	Short stress group – *Taraxacum*-dominant diet	ab	b
	Long stress group – *Salix*-dominant diet	bc	b
	Long stress group – *Cistus*-dominant diet	c	b
	Long stress group – *Taraxacum*-dominant diet	a	b
